# Correction : Epidemiology and outcomes of early-onset AKI in COVID-19-related ARDS in comparison with non-COVID-19-related ARDS: insights from two prospective global cohort studies

**DOI:** 10.1186/s13054-023-04487-6

**Published:** 2023-05-26

**Authors:** Bairbre A. McNicholas, Emanuele Rezoagli, Andrew J. Simpkin, Sankalp Khanna, Jacky Y. Suen, Pauline Yeung, Daniel Brodie, Gianluigi Li Bassi, Tai Pham, Giacomo Bellani, John F. Fraser, John Laffey, the CCCC Consortium, Tala Al-Dabbous, Huda Alfoudri, Mohammed Shamsah, Qamrah Alhadad, Matly Hanan, Subbarao Elapavaluru, Ashley Berg, Christina Horn, Ahmed Reda Mohamed Elsayed Abdelhalim, Amro Essam Amer, Cinderella Omar Rageh Elnaggar, Ahmed Ayman Hassan, Ali Abdelaziz, Mohamed Abdelhalim, Yehia Samir Shaaban Aly Orabi, Zinah A. Alaraji, Mo’nes R. Muhaisen, Lana Almasri, Dana Mustafa, Shaher Hamdan, Yousef Al-Saba’a, Zaina Dalloul, Mohammed Alkahlout, Hamza Jaber, Osama Aldabbourosama, Aliae A. R. Mohamed Hussein, Zarief Kamel Emad, Sarah Khaled, Nouralsabah Mohamed, Ebtisam Hassanin, Abdelhafeez Hamdi, Abdelrahman Ragab, Mohammed G. Azizeldin, Yunis Mayasi, Stephan Schroll, Dan Meyer, Jorge Velazco, Ludmyla Ploskanych, Wanda Fikes, Rohini Bagewadi, Marvin Dao, Haley White, Alondra Berrios Laviena, Ashley Ehlers, Maysoon Shalabi-McGuire, Trent Witt, Lorenzo Grazioli, Luca Lorini, E. Wilson Grandin, Jose Nunez, Tiago Reyes, Diarmuid O’Briain, Stephanie Hunter, Mahesh Ramanan, Julia Affleck, Hemanth Hurkadli Veerendra, Sumeet Rai, Josie Russell-Brown, Mary Nourse, Mark Joseph, Brook Mitchell, Martha Tenzer, Ryuzo Abe, Hwa Jin Cho, In Seok Jeong, Nadeem Rahman, Vivek Kakar, Helen Sun, Alison Hanley, Nicolas Brozzi, Omar Mehkri, Sudhir Krishnan, Abhijit Duggal, Stuart Houltham, Jerónimo Graf, Roderigo Diaz, Roderigo Orrego, Camila Delgado, Joyce González, Maria Soledad Sanchez, Michael Piagnerelli, Josefa Valenzuela Sarrazin, Gustavo Zabert, Lucio Espinosa, Paulo Delgado, Victoria Delgado, Diego Fernando Bautista Rincón, Angela Maria Marulanda Yanten, Melissa Bustamante Duque, Daniel Brodie, Khaled Abouelmagd, Alyaa Elhazmi, Abdullah Al-Hudaib, Jeff Javidfar, Maria Callahan, Andy Dong, Charles Crepy D’Orleans, M. Azhari Taufik, Elizabeth Yasmin Wardoyo, Margaretha Gunawan, Nurindah S. Trisnaningrum, Vera Irawany, Muhammad Rayhan, Mauro Panigada, Antonio Pesenti, Alberto Zanella, Giacomo Grasselli, Sebastiano Colombo, Chiara Martinet, Gaetano Florio, Massimo Antonelli, Simone Carelli, Domenico L. Grieco, Motohiro Asaki, Kota Hoshino, Leonardo Salazar, Mary Alejandra Mendoza Monsalve, John Laffey, Bairbre McNicholas, David Cosgrave, Minha Atif, Fadi Qutishat, Caoimhe Laffey, Michaeala Van Der Walt, Joseph McCaffrey, Allison Bone, Yusuff Hakeem, James Winearls, Mandy Tallott, David Thomson, Ivan Joubert, Christel Arnold-Day, Jenna Piercy, Richard van Zyl Smit, Malcom Miller, Lisa Seymour, Francois van Heyningen, Gilbert Teyangesikayi, David Fredericks, Ali Ait Hssain, Jeffrey Aliudin, Al-Reem Alqahtani, Khoulod Mohamed, Ahmed Mohamed, Darwin Tan, Joy Villanueva, Ahmed Zaqout, Ethan Kurtzman, Arben Ademi, Ana Dobrita, Khadija El Aoudi, Juliet Segura, Gezy Giwangkancana, Shinichiro Ohshimo, Javier Osatnik, Anne Joosten, Antoni Torres, Minlan Yang, Ana Motos, Carlos Luna, Francisco Arancibia, Virginie Williams, Alexandre Noel, Nestor Luque, Marina Fantini, Ruth Noemi Jorge García, Enrique Chicote Alvarez, Anna Greti, Adrian Ceccato, Angel Sanchez, Ana Loza Vazquez, Ferran Roche-Campo, Diego Franch-Llasat, Divina Tuazon, Marcelo Amato, Luciana Cassimiro, Flavio Pola, Francis Ribeiro, Guilherme Fonseca, Heidi Dalton, Mehul Desai, Erik Osborn, Hala Deeb, Antonio Arcadipane, Gennaro Martucci, Giovanna Panarello, Stefano Vitiello, Claudia Bianco, Giovanna Occhipinti, Matteo Rossetti, Raffaele Cuffaro, Sung-Min Cho, Glenn Whitman, Marwan El Sayed, Walaa Mokhtar, Eslam El-Shenawy, Hiroaki Shimizu, Naoki Moriyama, Jae-Burm Kim, Nobuya Kitamura, Johannes Gebauer, Toshiki Yokoyama, Abdulrahman Al-Fares, Sarah Buabbas, Esam Alamad, Fatma Alawadhi, Kalthoum Alawadi, Mohamed Ahmed Khalefa, Nourah Ahmad Abdulaziz Al Ajeel, Mohammad Fathy Aly, Abdullah Al-Saleh, Abdullah Naanouh, Alaa Mohammed Elshourbgy, Abdulrahman Al-Fares, Mohamed Yousef Gad, Rania Mohamed ElRazaz, Ibrahim Khadadah, Ahmed Mohammed Almumin, Hala Altarakma, Hasan Albannay, Mohammed Kh Alsaleh, Mahmoud Saad Abdallah Radwan, Islam Ahmed Saadallah, Hiro Tanaka, Satoru Hashimoto, Masaki Yamazaki, Tak-Hyuck Oh, Mark Epler, Cathleen Forney, Louise Kruse, Jared Feister, Joelle Williamson, Katherine Grobengieser, Eric Gnall, Sasha Golden, Mara Caroline, Timothy Shapiro, Colleen Karaj, Lisa Thome, Lynn Sher, Mark Vanderland, Mary Welch, Sherry McDermott, Matthew Brain, Sarah Mineall, Maria Unwin, Lixian Chen, Tarnya Trezise, Laurie McKeon, Dai Kimura, Luca Brazzi, Gabriele Sales, Giorgia Montrucchio, Tawnya Ogston, Dave Nagpal, Karlee Fischer, Roberto Lorusso, Bas van Bussell, Maria Elena De Piero, Silvia Mariani, Rajavardhan Rangappa, Rajesh Mohan Shetty, P. Sujin Rai, Argin Ganesan, Mariano Esperatti, Nora Angélica Fuentes, Maria Eugenia Gonzalez, Diarmuid O’Briain, Edmund G. Carton, Ayan Sen, Amanda Palacios, Deborah Rainey, Gordan Samoukoviv, Josie Campisi, Lucia Durham, Emily Neumann, Cassandra Seefeldt, Octavio Falcucci, Amanda Emmrich, Jennifer Guy, Carling Johns, Kelly Potzner, Catherine Zimmermann, Angelia Espinal, Nina Buchtele, Michael Schwameis, Andrea Korhnfehl, Roman Brock, Thomas Staudinger, Stephanie-Susanne Stecher, Michaela Barnikel, Sófia Antón, Alexandra Pawlikowski, Akram Zaaqoq, Lan Anh Galloway, Caitlin Merley, Alistair Nichol, Marc Csete, Luisa Quesada, Isabela Saba, Daisuke Kasugai, Hiroaki Hiraiwa, Taku Tanaka, Eva Marwali, Yoel Purnama, Santi Rahayu Dewayanti, Ardiyan, Dafsah Arifa Juzar, Debby Siagian, Yih-Sharng Chen, Mark Ogino, Prashant Nasa, Christina Matthew, Nimisha Abdul Majeed, Indrek Ratsep, Andra-Maris Post, Piret Sillaots, Anneli Krund, Merili-Helen Lehiste, Tanel Lepik, Frank Manetta, Effe Mihelis, Iam Claire Sarmiento, Mangala Narasimhan, Michael Varrone, Mamoru Komats, Julia Garcia-Diaz, Catherine Harmon, S. Veena Satyapriya, Amar Bhatt, Nahush A. Mokadam, Alberto Uribe, Alicia Gonzalez, Haixia Shi, Johnny McKeown, Joshua Pasek, Juan Fiorda, Marco Echeverria, Rita Moreno, Bishoy Zakhary, Marco Cavana, Alberto Cucino, Giuseppe Foti, Marco Giani, Benedetta Fumagalli, Davide Chiumello, Valentina Castagna, Andrea Dell’Amore, Paolo Navalesi, Hoi-Ping Shum, Alain Vuysteke, Asad Usman, Andrew Acker, Benjamin Smood, Blake Mergler, Federico Sertic, Madhu Subramanian, Alexandra Sperry, Nicolas Rizer, Erlina Burhan, Menaldi Rasmin, Ernita Akmal, Faya Sitompul, Navy Lolong, Bhat Naivedh, Simon Erickson, Peter Barrett, David Dean, Julia Daugherty, Antonio Loforte, Irfan Khan, Mohammed Abraar Quraishi, Olivia DeSantis, Dominic So, Darshana Kandamby, Jose M. Mandei, Hans Natanael, Eka YudhaLantang, Anastasia Lantang, Surya Oto Wijaya, Anna Jung, George Ng, Wing Yiu Ng, Pauline Yeung Ng, Shu Fang, Alexis Tabah, Megan Ratcliffe, Maree Duroux, Ahmed Alajeeli, Ali Tarhabat, Shingo Adachi, Shota Nakao, Pablo Blanco, Ana Prieto, Jesús Sánchez, Meghan Nicholson, Warwick Butt, Alyssa Serratore, Carmel Delzoppo, Pierre Janin, Elizabeth Yarad, Richard Totaro, Jennifer Coles, Bambang Pujo, Robert Balk, Andy Vissing, Esha Kapania, James Hays, Samuel Fox, Garrett Yantosh, Pavel Mishin, Saptadi Yuliarto, Kohar Hari Santoso, Susanthy Djajalaksana, Arie Zainul Fatoni, Masahiro Fukuda, Keibun Liu, Paolo Pelosi, Denise Battaglini, Juan Fernando Masa Jiménez, Diego Bastos, Sérgio Gaião, Desy Rusmawatiningtyas, Young-Jae Cho, Su Hwan Lee, Tatsuya Kawasaki, Laveena Munshi, Pranya Sakiyalak, Prompak Nitayavardhana, Mohamed Bashir Elagili, Talat Ahmed Abu Salem, Tamara Seitz, Rakesh Arora, David Kent, Daniel Marino, Swapnil Parwar, Andrew Cheng, Jennene Miller, Shigeki Fujitani, Naoki Shimizu, Jai Madhok, Clark Owyang, Hergen Buscher, Claire Reynolds, Olavi Maasikas, AleksanBeljantsev, Vladislav Mihnovits, Takako Akimoto, Mariko Aizawa, Kanako Horibe, Ryota Onodera, Carol Hodgson, Aidan Burrell, Meredith Young, Timothy George, Kiran Shekar, Niki McGuinness, Lacey Irvine, Brigid Flynn, Tomoyuki Endo, Kazuhiro Sugiyama, Keiki Shimizu, Eddy Fan, Kathleen Exconde, Shingo Ichiba, Muhannud Binnawara, Leslie Lussier, Gösta Lotz, Maximilian Malfertheiner, Lars Maier, Esther Dreier, Neurinda Permata Kusumastuti, Colin McCloskey, Al-Awwab Dabaliz, Tarek B. Elshazly, Josiah Smith, Konstanty S. Szuldrzynski, Piotr Bielański, Yusuff Hakeem, Keith Wille, Srinivas Murthy, Ken Kuljit S. Parhar, Kirsten M. Fiest, Cassidy Codan, Anmol Shahid, Mohamed Fayed, Timothy Evans, Rebekah Garcia, Ashley Gutierrez, Hiroaki Shimizu, Tae Song, Rebecca Rose, Suzanne Bennett, Denise Richardson, Giles Peek, Lovkesh Arora, Kristina Rappapport, Kristina Rudolph, Zita Sibenaller, Lori Stout, Alicia Walter, Daniel Herr, Nazli Vedadi, Robert Bartlett, Antonio Pesenti, Shaun Thompson, Julie Hoffman, Xiaonan Ying, Bailey Williams, Emely Sanchez, Chika Akwani, Ryan Kennedy, Muhammed Elhadi, Matthew Griffee, Mary Mone, Anna Ciullo, Yuri Kida, Ricard Ferrer Roca, JordI Riera, Sofia Contreras, Cynthia Alegre, Christy Kay, Irene Fischer, Elizabeth Renner, Hayato Taniguci, James Lee, Daniel Plotkin, Barbara Wanjiru Citarella, Laura Merson, Emma Hartley, Bastian Lubis, Takanari Ikeyama, Ameen Alhamad, Mohamed Fathi, Mohammed Maher Hadhoud, Hasan Alhouri, Ahmed Mechi, Mohammed Saleh Alyasiri, Muhammed Zainab Alghali Elsaid, Hamza Shahla, Balu Bhaskar, Jae-Seung Jung, Shay McGuinness, Glenn Eastwood, Sandra Rossi Marta, Fabio Guarracino, Stacy Gerle, Emily Coxon, Bruno Claro, Mahmoud Eleisawy, Hasnaa Osama, Daniel Loverde, Namrata Patil, Vieri Parrini, Angela McBride, Kathryn Negaard, Angela Ratsch, Ahmad Abdelaziz, Juan David Uribe, Adriano Peris, Mark Sanders, Dominic Emerson, Muhammad Kamal, Hamza Faida, Pedro Povoa, Roland Francis, Ali Cherif, Sunimol Joseph, Matteo Di Nardo, Micheal Heard, Kimberly Kyle, Ray A. Blackwell, Amel Ouyahia, Michael Piagnerelli, Patrick Biston, Hye Won Jeong, Reanna Smith, Yogi Prawira, Giorgia Montrucchio, Arturo Huerta Garcia, Nahikari Salterain, Bart Meyns, Muhammed Elnasser, Marsha Moreno, Rajat Walia, Amit Mehta, Annette Schweda, Melissa Williams, Emad Amkhatirah, Kyung Hoon Kim, Alexandra Assad, Estefania Giraldo, Wojtek Karolak, Martin Balik, Elizabeth Pocock, Akram Mohamed, Evan Gajkowski, Kanamoto Masafumi, Nicholas Barrett, Yoshihiro Takeyama, Sunghoon Park, Faizan Amin, Fina Meilyana Andriyani, Serhii Sudakevych, Janos Schnur, Angela Ratsch, Magdalena Vera, Rodrigo Cornejo, Patrícia Schwarz, Ana Carolina Mardini, Thais de Paula, Ary Serpa Neto, Andrea Villoldo, Alexandre Siciliano Colafranceschi, Alejandro Ubeda Iglesias, Juan Granjean, Lívia Maria Garcia Melro, Giovana Fioravante Romualdo, Diego Gaia, Helmgton Souza, Filomena Galas, Rafael Máñez Mendiluce, Alejandra Sosa, Ignacio Martinez, Hiroshi Kurosawa, Mohammad Badr Almoshantaf, Juan Salgado, Beate Hugi-Mayr, Eric Charbonneau, Vitor Salvatore Barzilai, Veronica Monteiro, Rodrigo Ribeiro de Souza, Michael Harper, Nidhal Siddig, Hiroyuki Suzuki, Celina Adams, Jorge Brieva, Almu’atasim Khamees, Fadi Graige, Moh Supriatna, George Nyale, Faisal Saleem Eltatar, Jihan Fatani, Husam Baeissa, Ayman A. L. Masri, Ahmed Rabie, Mok Yee Hui, Masahiro Yamane, Hanna Jung, Ayorinde Mojisola Margaret, Newell Nacpil, Katja Ruck, Rhonda Bakken, Claire Jara, Tim Felton, Samar Tharwat, Lorenzo Berra, Bobby Shah, Arpan Chakraborty, Monika Cardona, Gerry Capatos, Bindu Akkanti, Abiodun Orija, Harsh Jain, Asami Ito, Mohamed Muftah, Brahim Housni, Amer Aldhalia, Sennen Low, Koji Iihara, Joselito Chavez, Kollengode Ramanathan, Gustavo Zabert, Krubin Naidoo, Ian Seppelt, Marlice VanDyk, Sarah MacDonald, Shingo Ichiba, Wael Hafez, Randy McGregor, Teka Siebenaler, Hannah Flynn, Kristi Lofton, Toshiyuki Aokage, Bakar Kvirkvelia, Kazuaki Shigemitsu, Andrea Moscatelli, Giuseppe Fiorentino, Matthias Baumgaertel, Serge Eddy Mba, Jana Assy, Amelya Hutahaean, Holly Roush, Kay A. Sichting, Francesco Alessandri, Debra Burns, Taha Husayn Alkhubouli, Ahmad Nasrallah, Ahmed Rabie, Gavin Salt, Carl P. Garabedian, Jonathan Millar, Malcolm Sim, Adrian Mattke, Danny McAuley, Jawad Tadili, Tim Frenzel, Yaron Bar-Lavie, Aaron Blandino Ortiz, Jackie Stone, Alexis Tabah, Antony Attokaran, Michael Farquharson, Brij Patel, Derek Gunning, Kenneth Baillie, Safia Adem, Pia Watson, Kenji Tamai, Gede Ketut Sajinadiyasa, Dyah Kanyawati, Marcello Salgado, Assad Sassine, Bhirowo Yudo, Scott McCaul, Bongjin Lee, Sang Min Lee, Arnon Afek, Yoshiaki Iwashita, Hammad Fadlalmola, Bambang Pujo Semedi, Neurinda Permata Kusumastuti, Noureldin Mohamed Mansour, Jack Metiva, Nicole Van Belle, Ignacio Martin-Loeches, Mohammed Al-Sadawi, Cenk Kirakli, Al-Touny Shimaa, Abusalama Abdurraouf, Lenny Ivatt, Saad Moharam, Chia Yew Woon, Hyun Mi Kang, Timothy Smith, Erskine James, Nawar Al-Rawas, Abdulrahman Almjersah, Yudai Iwasaki, Hamza Ashour, Hussein Embarek, Kenny Chan King-Chung, Vadim Gudzenko, Beate Hugi-Mayr, Fabio Taccone, Fajar Perdhana, Yoan Lamarche, Joao Miguel Ribeiro, Nikola Bradic, Klaartje Van den Bossche, Oude Lansink, Gurmeet Singh, Gerdy Debeuckelaere, Henry T. Stelfox, Cassia Yi, Jennifer Elia, Thomas Tribble, Shyam Shankar, Raj Padmanabhan, Bill Hallinan, Luca Paoletti, Yolanda Leyva, Tatuma Fykuda, Jenelle Badulak, Jillian Koch, Lisa Janowaik, Amy Hackman, Deb Hernandez, Jennifer Osofsky, Katia Donadello, Aizah Lawang, Josh Fine, Benjamin Davidson, Andres Oswaldo Razo Vazquez, Ibrahim Abdehaleem

**Affiliations:** 1 Department of Anaesthesia and Intensive Care Medicine, School of Medicine, Clinical Sciences Institute, University of Galway, Galway University Hospital, Saolta Hospital Group, Galway, H91 YR71 Ireland; 2 School of Medicine, University of Galway, Galway, Ireland; 3 School of Medicine and Surgery, University of Milano-Bicocca, Monza, Italy; 4 Department of Emergency and Intensive Care, San Gerardo University Hospital, Monza, Italy; 5 Queensland University of Technology, Brisbane, Australia; 6 CSIRO Australian e-Health Research Centre AU, Herston, Australia; 7 University of Queensland, Brisbane, Australia; 8 Department of Medicine, The University of Hong Kong and Queen Mary Hospital, Hong Kong, Hong Kong China; 9 Department of Medicine, Columbia College of Physicians and Surgeons, and Center for Acute Respiratory Failure, New-York-Presbyterian Hospital, New York, NY USA; 10 Service de Médecine Intensive‑Réanimation, AP-HP, Hôpital de Bicetre, Hôpitaux Universitaires Paris-Saclay, Le Kremlin-Bicêtre, France; 11 UVSQ, Inserm U1018, Equipe d’Epidémiologie Respiratoire Intégrative, Université Paris-Saclay, Villejuif, France

**Correction : Critical Care (2023) 27:3** 10.1186/s13054-022-04294-5

Following publication of the original article [1], the authors identified that the collaborating authors part of the collaborating author group CCCC Consortium was missing.

The collaborating author group is available and included as Additional file 1 in this article.

## Supplementary Information

**Additional file 1.** List of collaborating authors in the CCCC Consortium.
